# Supplementary material for: Panoramic analysis of coronaviruses carried by representative bat species in Southern China to better understand the coronavirus sphere
Source: Nat Commun. 2023 Sep 8;14:5537. doi: 10.1038/s41467-023-41264-z (PMC10491624; doi:10.1038/s41467-023-41264-z)
Supplement: Supplementary file 1 — Supplementary Inforrnation [file 41467_2023_41264_MOESM1_ESM.pdf]

Supplementary information for "Panoramic Analysis of Coronaviruses Carried by Representative Bat Species in Southern China to Better Understand the Coronavirus Sphere"

Yelin Han[1, #], Panpan Xu[1, #], Yuyang Wang[1, #], Wenliang Zhao[1], Junpeng Zhang[5], Shuyi Zhang[5], Jianwei Wang[1], Qi Jin[1, 3, 4, *], Zhiqiang Wu[1, 2, 3, 4, *]

[1]NHC Key Laboratory of Systems Biology of Pathogens, Institute of Pathogen Biology, Chinese Academy of Medical Sciences & Peking Union Medical College, Beijing, China

[2]Key Laboratory of Respiratory Disease Pathogenomics, Chinese Academy of Medical Sciences & Peking Union Medical College, Beijing, China

[3]Key Laboratory of Pathogen Infection Prevention and Control (Peking Union Medical College), Ministry of Education, Beijing, China

[4]State Key Laboratory of Respiratory Health and Multimorbidity, Chinese Academy of Medical Sciences, Beijing, China

[5]College of Animal Science and Veterinary Medicine, Shenyang Agricultural University, Shenyang, China

[*]Corresponding authors: Dr. Zhiqiang Wu (wuzq2009@ipbcams.ac.cn); Dr. Qi Jin (zdsys@vip.sina.com).

This PDF file includes:

1. Supplementary Figures 1 to 8
2. Supplementary Tables 1 to 2

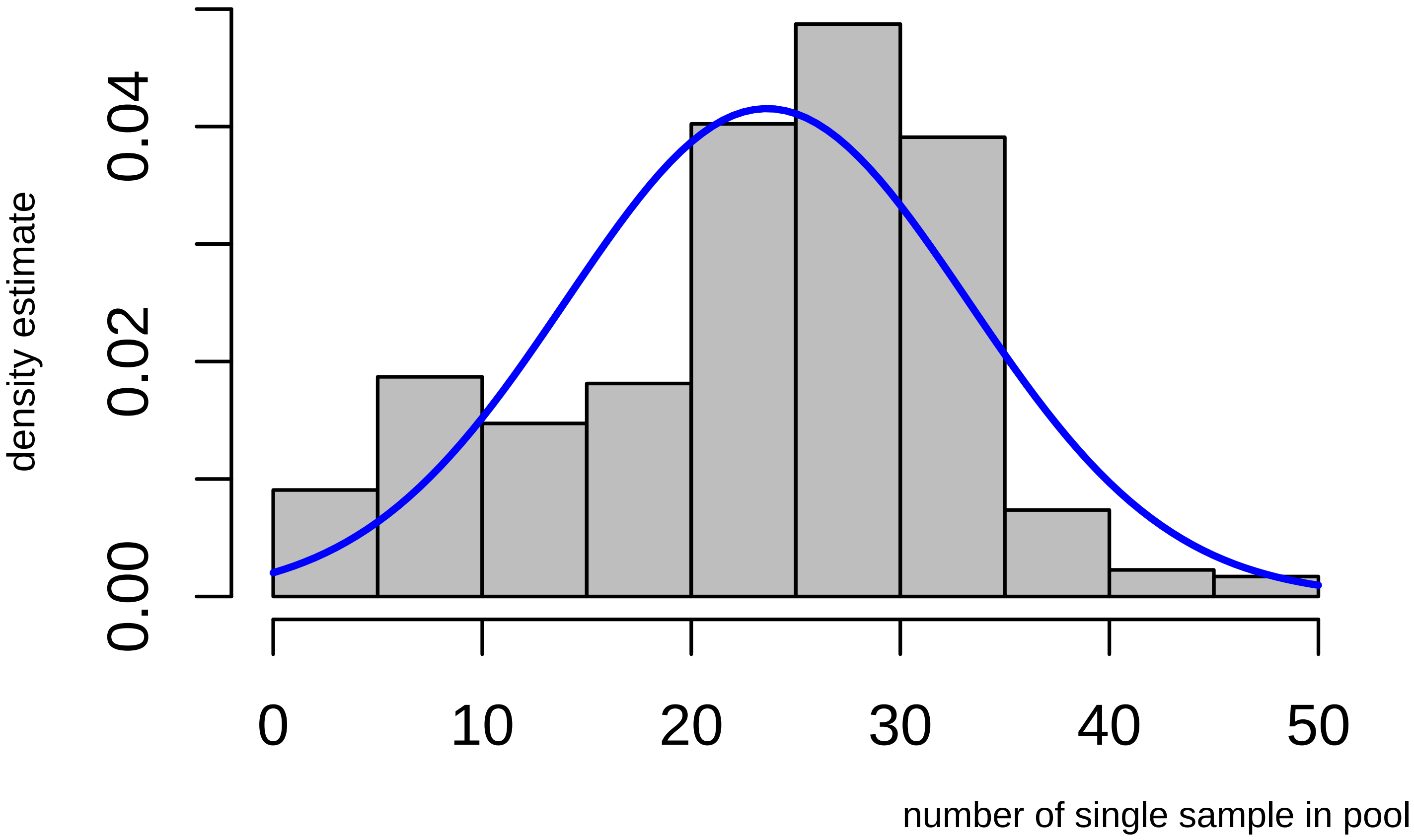

Supplementary Figure1: Probability Density Curve of Sample Pooling. The horizontal axis represents the number of individual samples involved in each pool in this study, and the vertical axis represents the probability density. The histogram represents the probability density corresponding to different numbers of individual samples in the pooled groups, generating the respective curves.

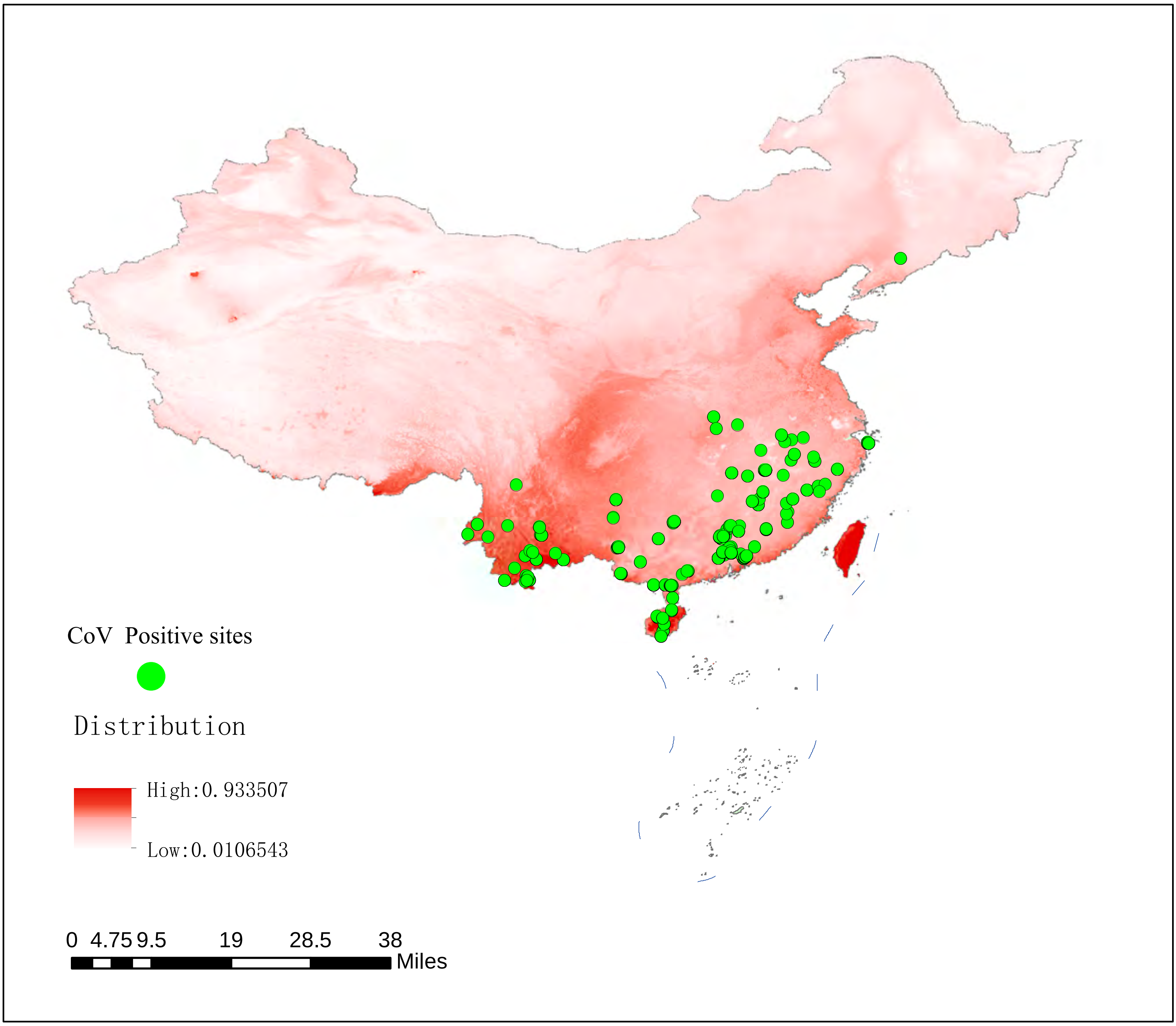

Supplementary Figure2: Distribution of bats and sampling sites for positive coronaviruses in China. The green dots in map represent sampling sites for positive coronaviruses from 2016 to 2021 and the color of the map represents the abundance of bats known to carry coronavirus in China.Map data were retrieved from 10.5281/zenodo.4167299.

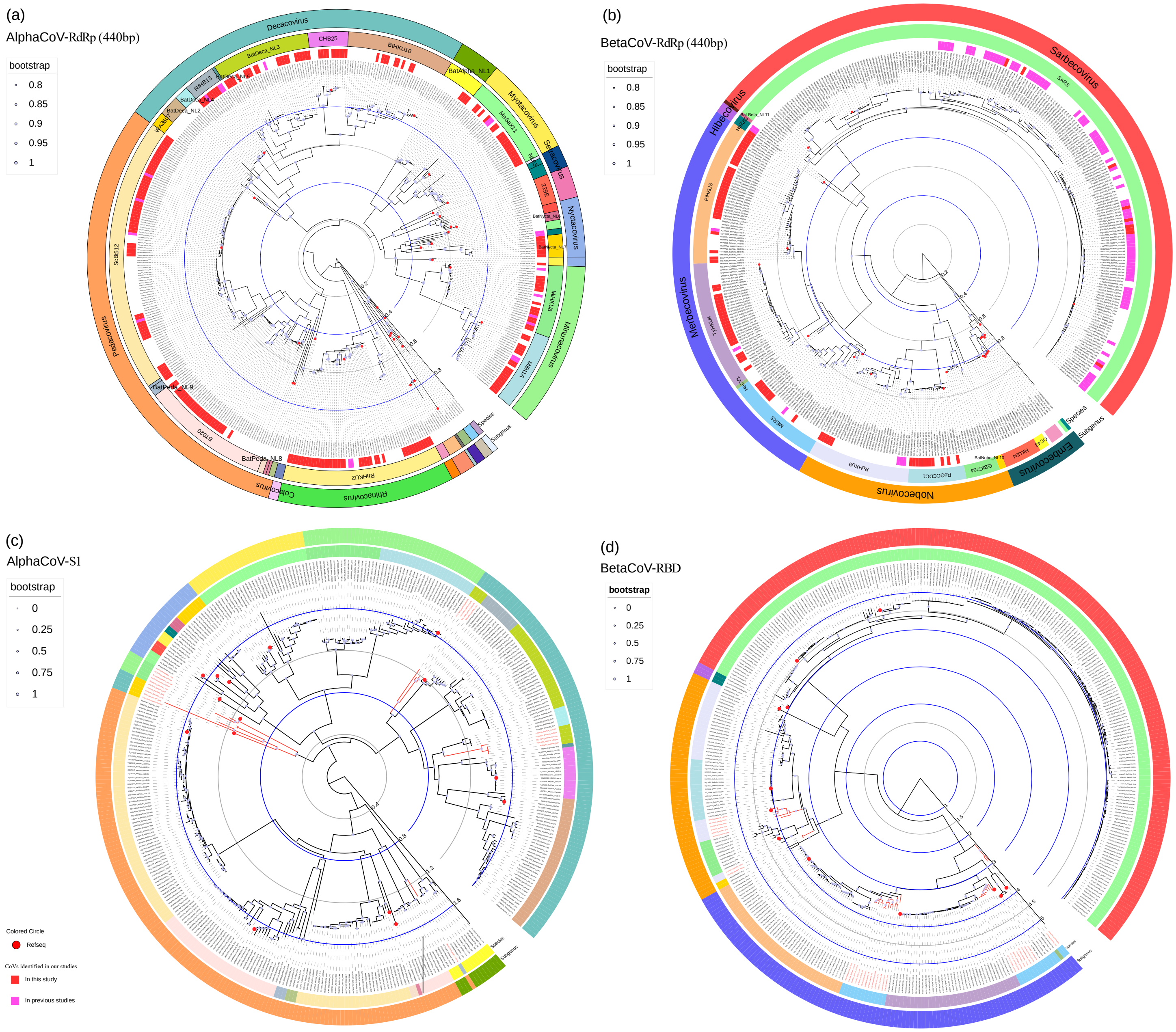

Virus-Subgenus	*Minunacovirus*		*Nyctacovirus*						*Decacovirus*								unclassified Alphacoronavirus
Virus-Species	MiBt1A	MiHKU8	PKBt3398	BatNycta_NL7	NvSC13	TyHKU33	WA2028	BatNycta_NL6	BatDeca_NL2	BatDeca_NL4	RfHB13	BatDeca_NL3	BatDeca_NL5	CHB25	BtHKU10	WA3607	BatAlpha_NL1

Virus-Subgenus	*Myotacovirus*	*Setracovirus*		*Duvinacovirus*	*Colacovirus*	*Pedacovirus*						*Luchacovirus*	*Rhinacovirus*	*Amalacovirus*	*Soracovirus*	*Sunacovirus*	*Minacovirus*	*Tegacovirus*
Virus-Species	MaSaX11	NL63	BtKYNL63	229E	CDPHE15	BatPeda_NL8	WA1087	PEDV	BT020	BatPeda_NL9	ScBt512	LuchengRn	RhHKU2	AMALF	SorexT14	SuncuX74	MinkCoV1	AlphaCoV1

Virus-Subgenus	*Embecovirus*					*Nobecovirus*				*Merbecovirus*				*Hibecovirus*	*Sarbecovirus*	unclassified Betacoronavirus
Virus-Species	Myodes2JL14	HKU1	MHV	HKU24	OC43	BatNobe_NL10	RoGCCDC1	RoHKU9	EiBtC704	HeCV1	TyHKU4	PiHKU5	MERS	HpZJ13	SARS	Bat Beta_NL11

Supplementary Figure3: Cluster dendrogram of Chinese provinces based on similarities between their mammalian diversity (hierarchical clustering). Provinces with CoV sequences available in this study highlighted in bold.Phylogenetic Trees of RdRp, S1 and RBD in Alpha-CoVs and Beta-CoVs. This figure presents the phylogenetic trees based on the RdRp (440bp), S1, and RBD sequences. The trees were constructed via the maximum likelihood method utilizing the GTR (RdRp) and IG (S1 and RBD) model. Each tree clearly denotes the subgenus, viral species within each subgenus, and their corresponding labels. (a) Phylogenetic tree of Alpha-CoVs using appropriate models (GTR+F+I+G4) by iqtree based on the RdRp sequence. (b) Phylogenetic tree of Beta-CoVs using appropriate models (TIM2+F+I+G4) by iqtree based on the RdRp sequence. (c) Phylogenetic tree of Alpha-CoVs based on the S1 sequences. (d) Phylogenetic tree of Beta-CoVs based on the RBD sequences.

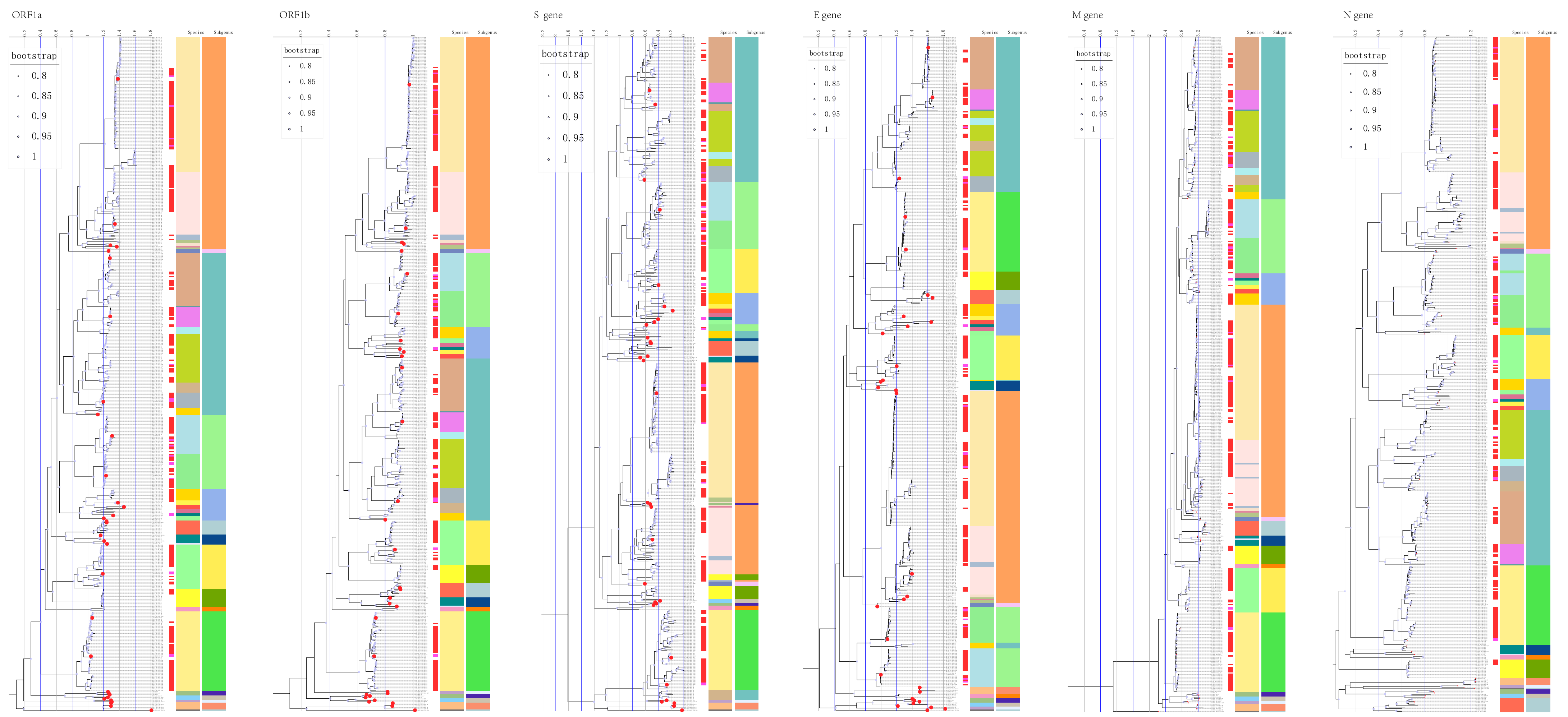

Virus-Subgenus	*Minunacovirus*		*Nyctacovirus*						*Decacovirus*								unclassified Alphacoronavirus
Virus-Species	MiBt1A	MiHKU8	PKBt3398	BatNycta_NL7	NvSC13	TyHKU33	WA2028	BatNycta_NL6	BatDeca_NL2	BatDeca_NL4	RfHB13	BatDeca_NL3	BatDeca_NL5	CHB25	BtHKU10	WA3607	BatAlpha_NL1

Virus-Subgenus	*Myotacovirus*	*Setracovirus*		*Duvinacovirus*	*Colacovirus*	*Pedacovirus*						*Luchacovirus*	*Rhinacovirus*	*Amalacovirus*	*Soracovirus*	*Sunacovirus*	*Minacovirus*	*Tegacovirus*
Virus-Species	MaSaX11	NL63	BtKYNL63	229E	CDPHE15	BatPeda_NL8	WA1087	PEDV	BT020	BatPeda_NL9	ScBt512	LuchengRn	RhHKU2	AMALF	SorexT14	SuncuX74	MinkCoV1	AlphaCoV1

Colored Circle
Refseq

CoVs identified in our studies
In this study
In previous studies

Supplementary Figure4: Phylogenetic tree based on ORF1a, ORF1b, S, E, M, N in alphacoronavirus. The trees were constructed by the maximum likelihood method using appropriate models (GTR) by FastTree. The subgenus, viral species of each subgenus and their corresponding labels are show on the phylogenetic tree in corresponding colors. The subgenus and viral species represented by different colors can be found in the table below.

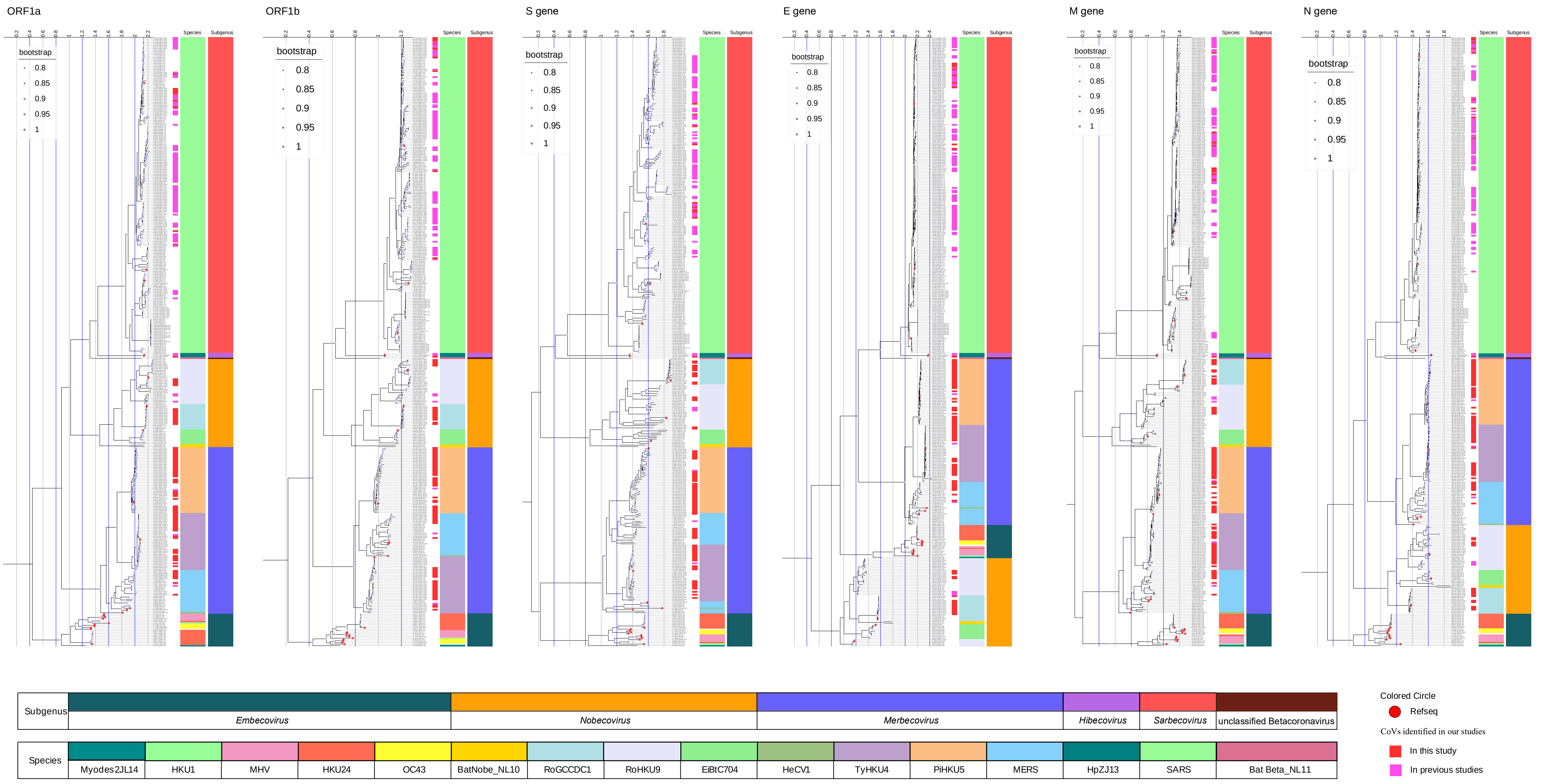

Supplementary Figure5: Phylogenetic tree based on ORF1a, ORF1b, S, E, M, N in betacoronavirus. The trees were constructed by the maximum likelihood method using appropriate models (GTR) by FastTree. The subgenus, viral species of each subgenus and their corresponding labels are show on the phylogenetic tree in corresponding colors. The subgenus and viral species represented by different colors can be found in the table below.

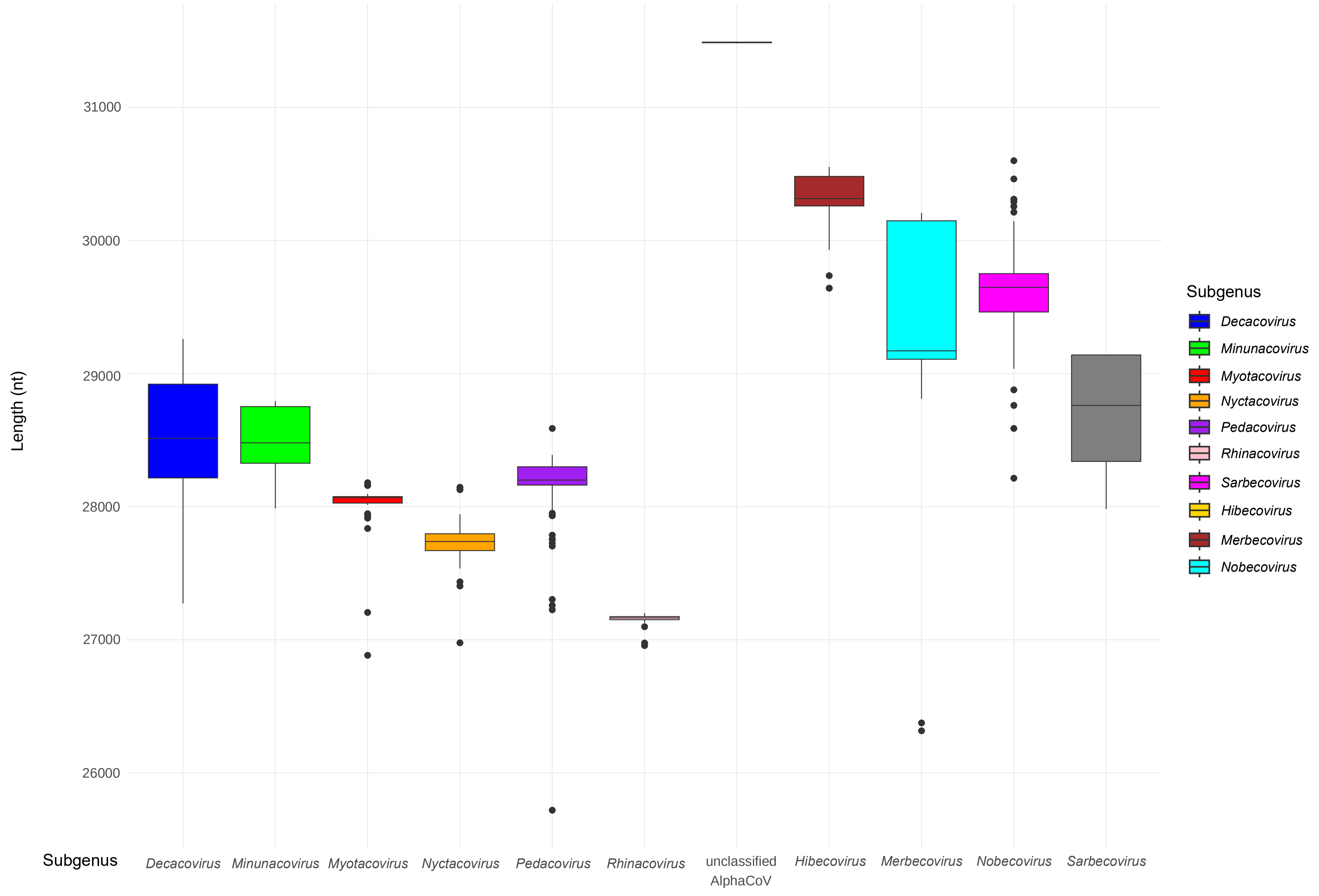

Supplementary Figure6: The generated box plot demonstrates the distribution of genome lengths across various coronavirus subgenera. The x-axis signifies different subgenera. On the y-axis, the genome length (nt) is depicted. Each subgenus's distribution is represented using box plots, showcasing the interquartile range (IQR) as the box's height, with the median line inside. Distinct subgenera are differentiated on the box plot by employing varying colors, as indicated in the legend located on the right-hand side of the figure.Whiskers extend to 1.5 times the IQR, encapsulating potential outliers exhibited as dots. This visualization provides valuable insights into genome size variations among the studied coronaviruses within specific subgenera.

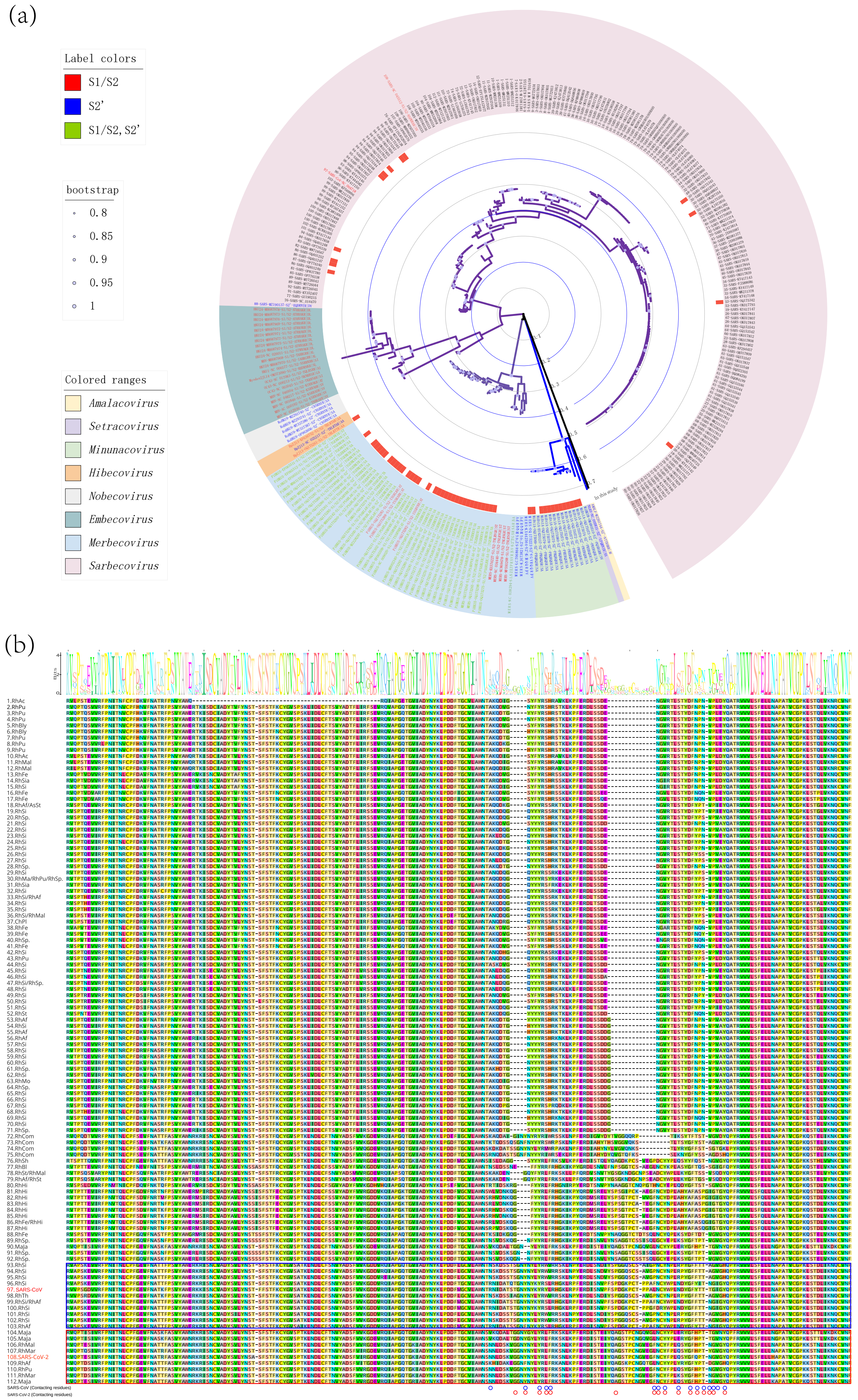

(a)
Label colors
S1/S2
S2'
S1/S2, S2'
bootstrap
0.8
0.85
0.9
0.95
1
Colored ranges
Amalacovirus
Setracovirus
Minunacovirus
Hibecovirus
Nobecovirus
Embecovirus
Merbecovirus
Sarbecovirus
In this study
(b)
SARS-CoV (Contacting residues)
SARS-CoV-2 (Contacting residues)

Supplementary Figure7: Detailed analysis of furin cleavage sites and Sarbecovirus RBDs. (a) A Maximum Likelihood phylogenetic tree constructed from all known S protein sequences of Sarbecovirus and CoVs that possess a furin cleavage site. Furin site details are presented in the respective tree labels. Distinct colored ranges denote different CoV subgenera. (b) Alignment of Sarbecovirus RBD amino acid sequences, after merging identical sequences. We have merged the strains with same RBD sequences. In total, there are 112 unique RBD sequences within our Sarbecovirus dataset. The name for the merged RBD sequences is as follows: the merge number followed by the types of species involved in the merged RBD sequences, represented by their corresponding species abbreviations. Additionally, in the phylogenetic tree shown in (a), for sequences in Sarbecovirus we have labeled the merged strains that are incorporated into the same RBD with their corresponding merge numbers. This means that strains in (a) with the same RBD amino acid sequences merged into the same RBD have the same preceding merge number. In addition, below the RBD alignment figure, we have utilized red and blue circles to respectively indicate the contacting residues of SARS-CoV-2 and SARS-CoV-2.

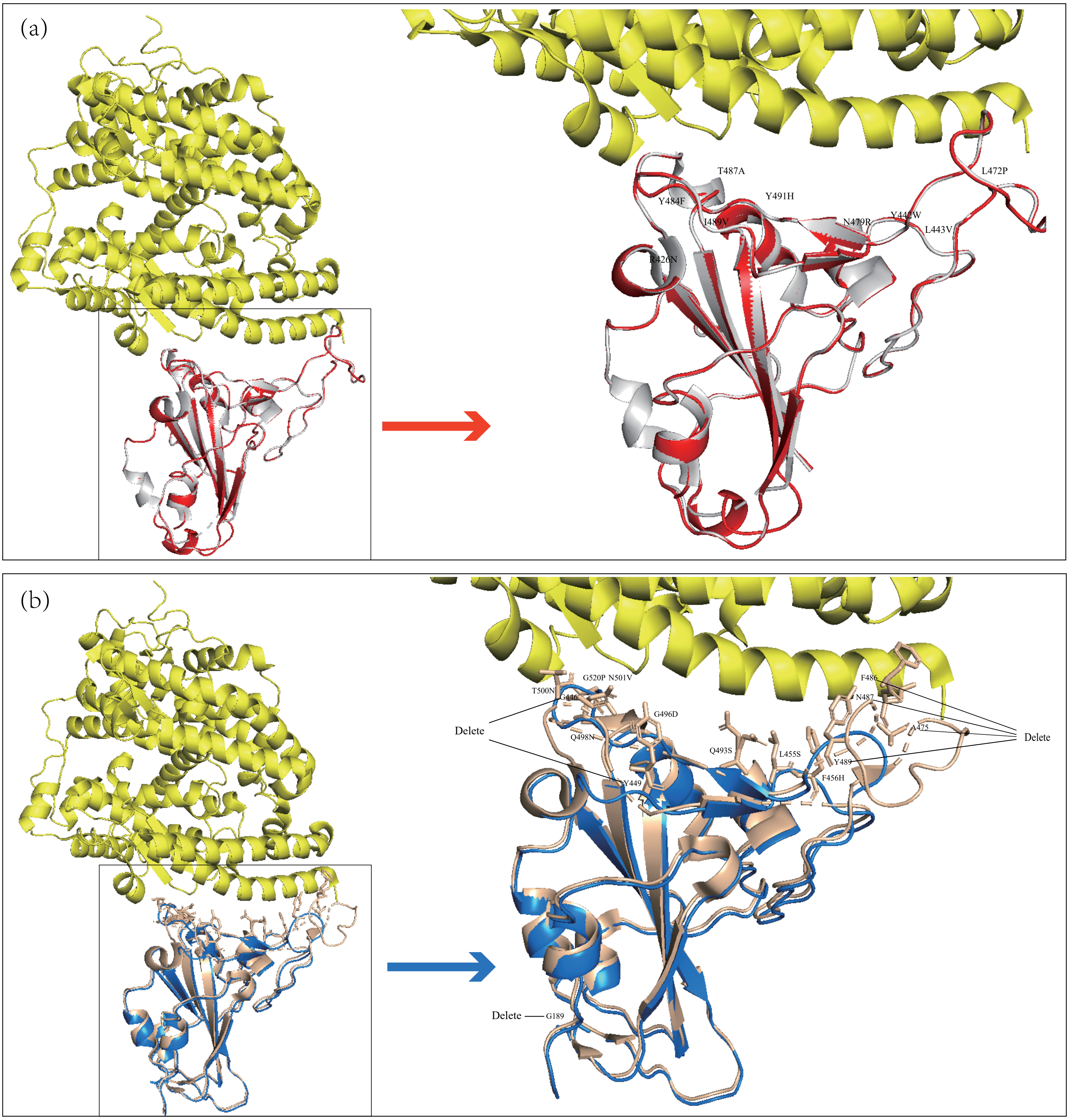

Supplementary Figure8: Homology modeling and structural comparison. Homology modeling and structural comparison of SARSr-CoV. (a) Structural comparison of YN2020B:hACE2 and SARS-CoV: hACE2. The amino acid residues that exhibited variations between the RBD of SARS-CoV and YN2020B were marked. RBD of YN2020B and SARS-CoV are colored red and gray, respectively. hACE2 is colored yellow. Homology modeling and structural comparison of SC2r-CoV. (b) Structural comparison of HN2021A:hACE2 and SARS-CoV-2: hACE2. The amino acid residues that exhibit variations between the RBD of SARS-CoV-2 and HN2021A were marked. RBD of HN2021A and SARS-CoV-2 are colored blue and gray, respectively. hACE2 is colored yellow.

Supplementary Table1: Host switches between bat genus for alpha-CoVs and their corresponding Bayes Factor (BF) and posterior probability (PP). Only switches with BF >3 are presented.

From	To	BF	PP
Myotis	Chalinolobus	72.5828924857	0.8617002751
Rhinolophus	Ozimops	417.1788997362	0.9728346045
Hipposideros	Mustela	592.1160301825	0.9807056037
Hipposideros	Pipistrellus	16.2384887573	0.5822798036
Hipposideros	Nyctalus	3.5160185651	0.2318462021
Megaderma	Tylonycteris	495.1476610132	0.9770138961
Megaderma	Suncus	3.9633894248	0.2538571379
Megaderma	Desmodus	6.5768453347	0.3608470031
Mops	Nyctalus	4448.1638577560	0.9973879427
Chaerephon	Murina	204.7064603701	0.9461567931
Aselliscus	Chalinolobus	10778.2212249146	0.9989203497
Ozimops	Nyctalus	334474.3365904890	1.0000000000
Ozimops	Scotophilus	334474.3365904890	1.0000000000
Ozimops	Eptesicus	3.1562184616	0.2131786995
Rousettus	Nyctalus	14.5561331044	0.5554626824
Cynopterus	Tylonycteris	16.0101839671	0.5788318880
Cynopterus	Vespadelus	497.4618023296	0.9771183784
Cynopterus	Nyctalus	334474.3365904890	1.0000000000
Miniopterus	Nyctalus	7105.0738165054	0.9983631108
Paguma	Gardnerycteris	3.3568134700	0.2236965834
Tylonycteris	Chalinolobus	2360.5917473103	0.9950893324
Tylonycteris	Nyctalus	334474.3365904890	1.0000000000
Tylonycteris	Triaenops	19.0854352965	0.6209730784
Tylonycteris	Eptesicus	3.7117223972	0.2416327099
Tylonycteris	Suncus	4.3770643911	0.2731167067
Tylonycteris	Desmodus	4.1911174238	0.2645839863
Afronycteris	Chalinolobus	43.2925632184	0.7879706056
Chalinolobus	Suncus	4.3227299928	0.2706439592
Chalinolobus	Desmodus	3.1699934659	0.2139100756
Nyctalus	Eptesicus	4.9266573026	0.2972172883

Supplementary Table2: Host switches between bat genus for beta-CoVs and their corresponding Bayes Factor (BF) and posterior probability (PP). Only switches with BF >3 are presented.

From	To	BF	PP
Myodes	Micropteropus	14395.4278610360	0.9992608024
Rattus	Micropteropus	3360.9860576008	0.9968416101
Hipposideros	Micropteropus	644.1727347854	0.9837376520
Hipposideros	Eonycteris	31682.7200197529	0.9996640011
Vespertilio	Eonycteris	158456.1958502320	1.0000000000
Plecotus	Hypsugo	712.9439606831	0.9852832471
Plecotus	Cynopterus	101.6592440971	0.9051811034
Plecotus	Eonycteris	79222.7734562351	0.9998656004
Pipistrellus	Eptesicus	20.2473079224	0.6553323029
Pipistrellus	Rhinolophus	5.1126340969	0.3243733620
Pipistrellus	Aselliscus	18.1004896809	0.6295947853
Eptesicus	Rhinolophus	4.9589459627	0.3177205833
Eptesicus	Pteropus	158456.1958502320	1.0000000000
Eptesicus	Aselliscus	494.0225423241	0.9788992675
Eptesicus	Chaerephon	3.6234970153	0.2538807876
Tylonycteris	Pteropus	158456.1958502320	1.0000000000
Rhinolophus	Aselliscus	5.5161997982	0.3412405080
Laephotis	Pteropus	93.4685685380	0.8977219273
Myotis	Rousettus	198.1347838304	0.9489953632
Myotis	Eonycteris	3.7244720649	0.2591223708
Io	Pteropus	2630.4651419104	0.9959680129
Miniopterus	Myonycteris	79.4914743868	0.8818627780
Rousettus	Eonycteris	3.7952189556	0.2627511592
Cynopterus	Aselliscus	56.7550794561	0.8420133056
Pteropus	Macroglossus	11.5889016786	0.5211343324
Pteropus	Micropteropus	2221.2784534901	0.9952288153
Pteropus	Chaerephon	18.7403137367	0.6376587595
Macroglossus	Micropteropus	6.9898471456	0.3962771319
Eidolon	Eonycteris	4.2866742452	0.2870102816
