# Supplementary material for: Panoramic analysis of coronaviruses carried by representative bat species in Southern China to better understand the coronavirus sphere
Source: Nat Commun. 2023 Sep 8;14:5537. doi: 10.1038/s41467-023-41264-z (PMC10491624; doi:10.1038/s41467-023-41264-z)
Supplement: Supplementary file 3 — Description of Additional Supplementary Files [file 41467_2023_41264_MOESM3_ESM.pdf]

Description of additional supplementary files

Supplementary data 1

Title: Sampling statistics on the species and region of the bat sample

Description: The dataset presents the number of bat samples collected from fourteen provinces in China, annotated with species, genus, and family information. It provides a statistical compilation of bat species totals across different taxonomic dimensions.

Supplementary data 2

Title: Bat CoV sequences obtained in 13064 bat samples, including 1310 in this study and 146 in previous studies

Description: The fundamental details of 1310 Bat CoV sequences obtained in this study, along with 146 Bat CoV sequences acquired previously, are comprehensively presented. This includes information such as Accession ID, Sequence label, Completeness, and more. Coupled with this information is the corresponding host information, geographical context, and sampling dates. Furthermore, the incorporation of each viral sequence within specific datasets is also elaborated upon.

Supplementary data 3

Title: Detection rate of CoVs from different bat species in different subgenera and provinces

Description: The present study portrays the detection rates of various subgenera of coronaviruses within distinct bat species across different provinces. The detection rate

of a specific subgenus within a bat species is calculated by dividing the number of samples identified as belonging to that subgenus, extracted from the respective bat species, by the total number of samples from that bat species. Similarly, the coronavirus detection rate of a specific bat species within a particular province is determined by dividing the count of coronavirus-positive samples from that bat species, collected in the respective province, by the total number of samples of that bat species gathered within that province.

Supplementary data 4

Title: Pairwise Comparisons of Host-Family

Description: Each row tests the null hypothesis that the host family 1 and host family 2 distributions are the same. Asymptotic significances (two-sided tests) are displayed. The significance level is 0.05. a. Significance values have been adjusted by the Bonferroni correction for multiple tests.

Supplementary data 5

Title: Pairwise Comparisons of Host-species

Description: Each row tests the null hypothesis that the host species 1 and host species 2 distributions are the same. Asymptotic significances (two-sided tests) are displayed. The significance level is 0.05. a. Significance values have been adjusted by the Bonferroni correction for multiple tests.

Supplementary data 6

Title: CoV sequences collected from public database

Description: The fundamental details of CoV sequences collected from public databases are comprehensively presented, including sequence metadata, data provenance, host information, sampling timestamps, and geographical coordinates. Additionally, the utilization of these sequences within the relevant datasets mentioned in the article is also delineated.

Supplementary data 7

Title: The identity between CoVs we identified and standard strains of different species in seven conserved regions of CoVs

Description: The identity between coronaviruses in this study and standard strains of different species is assessed across seven conserved regions. According to the classification criteria established by the International Committee on Taxonomy of Viruses (ICTV), instances where the identity falls below 90% are considered indicative of novel viral species.

Supplementary data 8

Title: The recombination events in the subgenera of alphacoronavirus and betacoronavirus

Description: Recombination events were detected utilizing the RDP5 software. The term "Minor Parent" refers to the parent contributing the smaller fraction of the

sequence, while "Major Parent" denotes the parent contributing the larger fraction of the sequence. "Unknown" signifies a situation where only one parent and a recombinant are present in the alignment, making a recombination event detectable. Sequences listed as "unknown" are used to infer the existence of a missing parental sequence. "NS" indicates that no significant P-value was recorded for this recombination event using the particular method in question. The terms "begin" and "end" correspond to the breakpoint positions in sequence alignment. The breakpoint positions labeled as "Begin" and "End" match the aligned positions of the sequences after alignment for each subgenus.

Supplementary data 9

Title: Hosts and sampled countries for alphacoronavirus and betacoronavirus viruses

Description: The alphacoronavirus and betacoronavirus viruses, along with their corresponding subgenus and virus species information, are delineated. Simultaneously, geographic details encompassing continent, region, country, and province, as well as host species, are summarized and compiled.

Supplementary data 10

Title: Furin site in different CoV Subgenera

Description: Sequences featuring Furin sites within distinct CoV subgenera, as identified through the ProP server (v1.0), are presented in a tabulated format. This presentation includes notations of Furin site positions, contextual information, and

corresponding scoring.
