# Supplementary material for: Panoramic analysis of coronaviruses carried by representative bat species in Southern China to better understand the coronavirus sphere
Source: Nat Commun. 2023 Sep 8;14:5537. doi: 10.1038/s41467-023-41264-z (PMC10491624; doi:10.1038/s41467-023-41264-z)
Supplement: Supplementary file 14 — Reporting Summary [file 41467_2023_41264_MOESM14_ESM.pdf]

nature portfolio

Corresponding author(s): Zhiqiang Wu, Qi Jin

Last updated by author(s): Aug 14, 2023

Reporting Summary

Nature Portfolio wishes to improve the reproducibility of the work that we publish. This form provides structure for consistency and transparency in reporting. For further information on Nature Portfolio policies, see our Editorial Policies and the Editorial Policy Checklist.

Statistics

For all statistical analyses, confirm that the following items are present in the figure legend, table legend, main text, or Methods section.

n/a	Confirmed	
[ ]	[x]	The exact sample size (*n*) for each experimental group/condition, given as a discrete number and unit of measurement
[ ]	[x]	A statement on whether measurements were taken from distinct samples or whether the same sample was measured repeatedly
[ ]	[x]	The statistical test(s) used AND whether they are one- or two-sided<br>*Only common tests should be described solely by name; describe more complex techniques in the Methods section.*
[x]	[ ]	A description of all covariates tested
[x]	[ ]	A description of any assumptions or corrections, such as tests of normality and adjustment for multiple comparisons
[ ]	[x]	A full description of the statistical parameters including central tendency (e.g. means) or other basic estimates (e.g. regression coefficient) AND variation (e.g. standard deviation) or associated estimates of uncertainty (e.g. confidence intervals)
[ ]	[x]	For null hypothesis testing, the test statistic (e.g. *F*, *t*, *r*) with confidence intervals, effect sizes, degrees of freedom and *P* value noted<br>*Give P values as exact values whenever suitable.*
[ ]	[x]	For Bayesian analysis, information on the choice of priors and Markov chain Monte Carlo settings
[x]	[ ]	For hierarchical and complex designs, identification of the appropriate level for tests and full reporting of outcomes
[x]	[ ]	Estimates of effect sizes (e.g. Cohen's *d*, Pearson's *r*), indicating how they were calculated

Our web collection on statistics for biologists contains articles on many of the points above.

Software and code

Policy information about availability of computer code

Data collection	Reference genome sequence data were downloaded from GenBank and GISAID using the web interface.
Data analysis	Software used: MEGAN6, Geneious Prime, MAFFT v7.475, DNA Star package, FastTree, Iqtree, Figtree, Interactive Tree Of Life, ProP server, SnapGene, RDP5, BEAST, Tracer v1.7.154, Breakpoint Distribution Plot, SpreaD3, Gephi, GARD, R 4.2.3, pheatmap (R package) 1.0.12, vegan (R package) 2.6.4, ggplot2 (R package) 3.4.2.

For manuscripts utilizing custom algorithms or software that are central to the research but not yet described in published literature, software must be made available to editors and reviewers. We strongly encourage code deposition in a community repository (e.g. GitHub). See the Nature Portfolio guidelines for submitting code & software for further information.

Data

Policy information about availability of data

All manuscripts must include a data availability statement. This statement should provide the following information, where applicable:

- Accession codes, unique identifiers, or web links for publicly available datasets
- A description of any restrictions on data availability
- For clinical datasets or third party data, please ensure that the statement adheres to our policy

The raw data, and CoV sequence generated in this study have been deposited in the Sequence Read Archive (SRA) and Genome Sequence Archive (GSA) of the National Genomics Data Center, respectively, under accession codes PRJNA994658 [https://www.ncbi.nlm.nih.gov/bioproject/PRJNA994658] and PRJCA009015 [https://ngdc.cncb.ac.cn/bioproject/browse/PRJCA009015]. The CoV sequences generated in this study have been deposited in the NCBI GenBank with accession

numbers OQ175021-OQ176213. DNA barcoding data from selected bat samples, targeting the mitochondrial cytochrome b gene, have been deposited in NCBI GenBank under accession numbers ON640659-ON640727. The protein structure data used in this study are available in the PDB database under accession code 2AJF.1 [http://doi.org/10.2210/pdb2AJF/pdb] and 6M0J [http://doi.org/10.2210/pdb6M0J/ pdb]. The Supplementary data 1-10 are archived in Figshare, accessible via the link [https://figshare.com/s/7460ae3960c82e9f3de0] with the associated DOI [10.6084/m9.figshare.23941539].

Research involving human participants, their data, or biological material

Policy information about studies with human participants or human data. See also policy information about sex, gender (identity/presentation), and sexual orientation and race, ethnicity and racism.

Reporting on sex and gender	N/A
Reporting on race, ethnicity, or other socially relevant groupings	N/A
Population characteristics	N/A
Recruitment	N/A
Ethics oversight	N/A

Note that full information on the approval of the study protocol must also be provided in the manuscript.

Field-specific reporting

Please select the one below that is the best fit for your research. If you are not sure, read the appropriate sections before making your selection.

☒ Life sciences ☐ Behavioural & social sciences ☐ Ecological, evolutionary & environmental sciences

For a reference copy of the document with all sections, see nature.com/documents/nr-reporting-summary-flat.pdf

Life sciences study design

All studies must disclose on these points even when the disclosure is negative.

Sample size	The sample size for this study, encompassing 13,064 bat samples spanning 54 bat species, 19 genera, and seven families, was not determined using formal statistical power calculations. Rather, it was based on a comprehensive approach to achieve a broad and representative overview of the CoV diversity across various bat species and regions in China.
Data exclusions	No data were excluded.
Replication	There was no separation of experimental groups in the study, hence no replication.
Randomization	There was no separation of experimental groups in the study, hence no randomization.
Blinding	There was no separation of experimental groups in the study, hence no blinding.

Reporting for specific materials, systems and methods

We require information from authors about some types of materials, experimental systems and methods used in many studies. Here, indicate whether each material, system or method listed is relevant to your study. If you are not sure if a list item applies to your research, read the appropriate section before selecting a response.

Materials & experimental systems

n/a	Involved in the study	
☒	☐	Antibodies
☒	☐	Eukaryotic cell lines
☒	☐	Palaeontology and archaeology
☐	☒	Animals and other organisms
☒	☐	Clinical data
☒	☐	Dual use research of concern
☒	☐	Plants

Methods

n/a	Involved in the study	
☒	☐	ChIP-seq
☒	☐	Flow cytometry
☒	☐	MRI-based neuroimaging

Animals and other research organisms

Policy information about studies involving animals; ARRIVE guidelines recommended for reporting animal research, and Sex and Gender in Research

Laboratory animals	No laboratory animals were involved in the study.
Wild animals	Bats were captured in their natural habitats, which included karstic caves, forests, woods, or abandoned buildings, employing a combination of hand nets (for catching bats in caves or buildings), mist nets (for sealing the cave or building exits), and harp traps (for trapping bats in forests or Following capture, each bat was stored in a cotton bag for swab collection. Pharyngeal and anal swabs were collected from live bats. After sample collection, the bats were released.
Reporting on sex	N/A. Sex was not considered in the study design.
Field-collected samples	Field-collected oral and anal swabs samples from bats were involved in the study. Pharyngeal and anal swabs were collected from live bats, with samples immediately immersed in virus sampling tubes (Yocon, Beijing, China) containing maintenance medium. These were initially stored in a portable cooler at −20°C or in liquid nitrogen for temporary preservation during fieldwork. Subsequently, samples were transferred to our laboratory and stored at −80 °C for long-term preservation.
Ethics oversight	Animals were treated according to the guidelines of the Regulations for the Administration of Laboratory Animals (Decree No. 2 of the State Science and Technology Commission of the People's Republic of China, 1988). Sampling procedures were approved by the Ethics Committee of the Institute of Pathogen Biology, Chinese Academy of Medical Sciences & Peking Union Medical College (Approval number: IPB EC20100415)

Note that full information on the approval of the study protocol must also be provided in the manuscript.
